# An expert opinion on the management of pediatric patients with wheezing and mild asthma: translating 2025 GINA strategy report into clinical practice in Italy

**DOI:** 10.1186/s13052-026-02206-9

**Published:** 2026-02-04

**Authors:** Francesca Santamaria, Eugenio Baraldi, Luca Cavalieri, Renato Cutrera, Stefania La Grutta, Giorgio Piacentini, Gherardo Siscaro

**Affiliations:** 1 Department of Translational Medical Sciences, Pediatric Pulmonology, Federico II University, Naples, Italy; 2 Department of Women’s and Children’s Health, University Hospital of Padova, Padua, Italy; 3 Institute of Pediatric Research, “Città della Speranza”, Padua, Italy; 4 Medical Doctor, Consultant, Chiesi Italia S.p.A, Parma, Italy; 5 Pneumology and Cystic Fibrosis Unit, Bambino Gesù’ Children’s Hospital, IRCCS, Rome, Italy; 6 Institute of Translational Pharmacology, National Research Council, Palermo, Italy; 7 Department of Surgical Sciences, Dentistry, Gynecology and Pediatrics, University of Verona, Verona, Italy; 8 Medical Affairs Department, Chiesi Italia S.p.A., Parma, Italy

**Keywords:** Preschool wheezing, Pediatric asthma, Mild asthma, Pre-asthma, GINA 2025, Inhaled corticosteroids, Inhalant therapy, Caregiver education

## Abstract

**Background:**

Preschool wheezing and mild asthma are among the most frequent chronic respiratory conditions in children. These conditions can significantly affect quality of life, be associated with wheeze exacerbations, and predispose to persistent airway disease. Early recognition and tailored management are crucial to prevent long-term morbidity.

**Objective:**

This Expert Opinion paper aims to translate the 2025 Global Initiative for Asthma (GINA) Strategy Report into the Italian clinical context, providing guidance for early recognition, risk stratification, and personalized management of pediatric patients with wheezing and mild asthma.

**Content:**

The paper discusses the heterogeneity of preschool wheezing phenotypes and the emerging concept of “pre-asthma”, highlighting the prognostic role of biomarkers such as blood eosinophils, fractional exhaled nitric oxide (FeNO), and early aeroallergen sensitization in predicting disease persistence. It also addresses the impact of early-life wheezing on lung function trajectories, challenges in defining mild asthma, and the rationale for stepwise treatment strategies according to age. Particular emphasis is placed on practical aspects of care, such as the use of low dose of inhaled corticosteroids (ICS), suboptimal adherence to therapy, inhaler misuse, the benefits of dose counters to avoid pseudo-adherence, and the importance of caregiver education and shared decision-making in inhaler selection.

**Summary:**

Applying GINA 2025 principles through individualized, biomarker-informed, and education-based strategies can improve outcomes in children with early or mild asthma, bridging the gap between evidence-based recommendations and usual pediatric care in Italy.

**Supplementary Information:**

The online version contains supplementary material available at 10.1186/s13052-026-02206-9.

## Introduction

From a clinical perspective, asthma is a chronic disorder which can occur at any age [1]. However, many patients show signs of airway issues already in infancy, suggesting that asthma likely originates early in life [2].

Three phases have been proposed to explain the development of asthma in childhood: (a) *susceptibility to asthma* – which results in the onset of wheezing in the first months of life and is influenced by genetic, environmental, or socio-economic factors; (b) *pre-asthma* – a occurring between 1 and 3 years of age, in which children present mild, probably intermittent airway inflammation insufficient to cause overt symptoms, but can progress to asthma; and (c) *persistent asthma* – appearing from ages 4–6 years onward, mainly characterized by Type 2 eosinophilic and, to a lesser extent, Type 2–low inflammation [3, 4].

Preschool wheezing, particularly when recurrent or associated with atopy, increases the risk of later asthma [5]. Mild asthma, despite being considered a benign condition, accounts for a substantial proportion of morbidity with 30–40% of all asthma exacerbations occurring in this group [6]. Most of the asthmatics seen in the pediatric primary care have intermittent symptoms that do not constantly impact their lives [7]. However, school absenteeism, disturbed sleep, impaired quality of life, and progression to obstructive airway disease are reported in about two-thirds of children and adolescents with mild asthma, primarily because of insufficient prevention or treatment [8–10].

In the 1990s, the network Global Initiative for Asthma (GINA) was established for sharing information on asthma care, including children and adolescents [11]. The GINA Report and its annual updates have been translated into multiple languages and adopted worldwide. However, despite providing evidence-based guidance, applying GINA recommendations in everyday pediatric practice is challenging because of the different patterns of wheezing over time and the variable progression of the disease [12]. Furthermore, variations in medication use, inhaler costs and availability, as well as differences in regulatory approvals of anti-asthma drugs across countries, restrict the universal implementation of the strategies recommended in the annual GINA Reports.

In Italy, an expert opinion group was convened to adapt the management of pediatric asthma to the national context, in alignment with GINA recommendations (https://ginasma.it/) [13]. In this document, efforts have been made to outline most of the key challenges encountered in the Italian setting when managing preschool wheezing as well as mild asthma. These include patients’ early identification, risk stratification and personalized treatment approaches, with special attention to the use of appropriate drug delivery systems. Our primary goal is to promote the application of GINA 2025 recommendations in real-world pediatric settings in Italy, thereby contributing to improved long-term disease control and overall patient outcomes.

## Preschool wheezing

### Early clinical evaluation and phenotypic characterization

Early evaluation of wheezing in preschool children is essential, as wheezing should not be considered a diagnosis. Multiple conditions can underlie this clinical manifestation, and management based solely on a presumptive diagnosis is inadequate. Differential diagnosis should consider structural and congenital anomalies (e.g., tracheobronchomalacia, vascular rings, tracheal or bronchial compression), persistent infections such as *Mycoplasma* or *Chlamydia*, gastroesophageal reflux, foreign body aspiration, prematurity-related lung disease including bronchopulmonary dysplasia, cystic fibrosis, tuberculosis, and ciliary dysfunction [14, 15]. Phenotypic assessment is relatively important in the evaluation of preschool wheezing. Despite the variety of described phenotypes, a practical distinction between transient wheezing and early-onset asthma would remain of relative importance. Approximately one-third of children experience wheezing within the first three years of life, although only a subset develops persistent asthma later on [14, 15].

Preschool wheezing represents a heterogeneous group of conditions with distinct phenotypic and endotypic profiles, linking clinical patterns to underlying molecular mechanisms. The main phenotypes—transient early, late-onset, and persistent wheeze—are typically defined retrospectively based on symptom timing and duration [15]. Transient early wheeze begins before age 3 and typically resolves by school age without lung function impairment; late-onset wheeze appears after age 3 and is often atopy-related, with reduced lung function and increased bronchial hyperresponsiveness; persistent wheeze starts early, frequently coexists with atopy and elevated IgE, and is associated with lung function decline by school age [16].

Because retrospective phenotypes have limited clinical applicability, the European Respiratory Society (ERS) proposes a pragmatic classification distinguishing episodic viral wheezing, which is mainly triggered by viral infections and tends to resolve with age, from multiple-trigger wheezing, which occurs in response to allergens, irritants, or exercise and is more often associated with atopy and persistent asthma [17]. Recently, the ERS Task Force agreed that more than one episode of wheezing is required in this age group (< 6 years), and that the term “recurrent preschool wheezing” provides greater clarity [17].

Therapeutic response may provide supporting evidence for the diagnosis: children with asthma-like phenotypes typically respond to inhaled corticosteroids (ICS), while those with transient viral wheezing may respond less to anti-inflammatory treatment. GINA recommend an 8–12-week ICS trial in children with recurrent or persistent wheeze to assess response to ICS-based maintenance treatment [11]. Clinical studies confirm that ICS improves symptoms and reduces exacerbations in preschool wheeze [18].

Integrating phenotypic assessment, risk factors, and treatment response helps clinicians differentiate benign wheezing from early asthma, allowing timely, personalized interventions to modify disease progression [19].

### Pre-asthma and risk factors progression from wheezing to asthma

The transition from recurrent wheezing to established asthma remains a major diagnostic challenge in preschool children. The emerging concept of “pre-asthma” describes an early, potentially reversible stage characterized by recurrent respiratory symptoms and Type 2 biomarkers [e.g., blood eosinophils (EBC), fractional exhaled nitric oxide (FeNO)], identifying children at high risk for persistent asthma [3, 4]. Key predictive factors include frequency and severity of wheezing episodes, atopy, prematurity, maternal asthma severity, and even the seasonal onset of symptoms—particularly those beginning in September, which have been associated with higher risk due to viral and allergen exposure [20, 21]. This stage offers a crucial window for early recognition and intervention, although mechanisms underlying progression remain partly unclear.

The Asthma Predictive Index (API) and its modified version (mAPI) are validated tools for identifying preschool children at increased risk of persistent asthma [22, 23]. They combine clinical and laboratory criteria to help distinguish transient wheezers from those likely to develop persistent asthma, guiding early interventions. While a positive result strongly predicts future asthma, a negative result does not reliably rule it out due to limited sensitivity [24]. More recently, advanced risk stratification models, incorporating machine learning and artificial intelligence, have been developed to integrate clinical, biological, and environmental data, enhancing predictive accuracy for personalized asthma risk assessment [25].

Clinical trajectories and symptom patterns in preschool wheezing—influenced by genetics, early-life infections, environmental exposure, parental asthma, and eosinophil levels—help identify children at risk of developing persistent asthma, while endotyping using biomarkers and infection profiles further refines risk by distinguishing atopic, non-atopic, and neutrophilic clusters in severe wheeze [26, 27].

The 2025 GINA report emphasizes evaluating both recurrence and severity of asthma-like symptoms in preschoolers, supporting an integrated approach that combines clinical assessment, biomarkers, and predictive models to optimize early recognition and guide management strategies [11].

### Biomarkers for early identification of asthma

Early identification of asthma risk in children, particularly in the preschool age group, is challenging due to the heterogeneous nature of wheezing disorders and the evolving immune system. Type 2 inflammatory biomarkers, including EBC [28–30], FeNO [31–33] and aeroallergen sensitization [34, 35] have emerged as valuable tools for risk stratification and guiding individualized monitoring and treatment. In early childhood, biomarkers should be interpreted dynamically, taking into account serial measurements, age-appropriate reference ranges, symptom patterns and predictive risk scores (Table 1). Given the limited sensitivity and specificity of individual biomarkers in children under 6 years, an integrated approach combining clinical assessment, serial biomarker measurements, and multivariable predictive tools such as API, modified API, and Pediatric Asthma Risk Score (PARS) provides superior accuracy for early asthma risk stratification and targeted intervention [31, 50, 51].

**Table 1:** Biomarkers for early asthma risk stratification: suggested Cut-offs, Interpretation, and caveats

Biomarker	Suggested pediatric cut-off(s)	Typical interpretation	Main caveats
FeNO (ppb)	**< 20**: low/less likely T2; **20–35**: intermediate; **> 35**: supportive of eosinophilic airway inflammation in children (some studies use 20–25 as lower threshold; others use ≥ 35).	Elevated FeNO supports Type-2 inflammation and predicts steroid responsiveness; useful for monitoring.	Cut-offs vary by age/atopy/device; preschool utility lower and thresholds may be lower – interpret clinically.
Blood eosinophils (cells/µL)	**< 150**: low; **≥ 150–300**: suggestive T2; **> 300**: increased risk in preschool wheezers; **≥ 400**: higher risk of exacerbations in some cohorts.	Higher counts associate with Type-2 phenotype, greater exacerbation risk, and potential ICS responsiveness.	Absolute thresholds vary; consider recent meta-analyses – preschool data highlight > 300 cells/µL as a risk marker.
Skin prick test / sIgE	Any clinically relevant sensitization (positive SPT or sIgE > laboratory cut-off)	Presence of sensitization increases risk of persistent asthma and atopic phenotype.	Predictive value depends on allergen, age, and local prevalence; single positive test is not definitive – combine with history.
Combined/Integrated approach	n/a (use combined markers + clinical risk score)	Combining clinical features (e.g., recurrent wheeze, eczema, parental asthma) with FeNO, eosinophils and sensitization yield better prediction than any single marker.	Recommended especially in preschool children – use multivariable risk tools (e.g., PARS or similar).

Abbreviations: FeNO: Fractional exhaled Nitric Oxide; SPT: Skin Prick Test; sIgE: specific Immunoglobulin E; ICS: inhaled corticosteroids; PARS: Pediatric Asthma Risk Score

### Early-life wheezing and its impact on lung function trajectories

Objective assessment of early lung function alterations is crucial. In preschool children, impulse oscillometry reliably measures respiratory resistance and reactance without forced manoeuvres, with elevated resistance at 5 Hz associated with reduced lung function at early school age [36]. In older children, spirometry remains the gold standard, showing lower FEV₁, FEV₁/FVC, and mid-expiratory flows (FEF₂₅–₇₅) in recurrent wheezers compared with peers [37]. Longitudinal studies reveal distinct lung function trajectories from childhood to early adulthood—including persistent, worsening obstruction or improvement—partly influenced by perinatal and early-life factors such as prematurity, in utero exposures (maternal smoking, nutrition, inflammation), and early respiratory illnesses [38].

Frequent exacerbations in early childhood are associated with accelerated lung function decline [39]. In those with severe wheezing, early airway remodeling contributes to persistent symptoms and impaired lung development, with a bidirectional relationship between structural changes and recurrent inflammation [40]. While no therapies currently prevent or reverse these changes, proactive management aimed at reducing exacerbations, particularly more than two severe episodes per year, remains vital to preserve long-term pulmonary function [39].

## Mild asthma: still a challenge for pediatricians?

Mild asthma in children is a heterogeneous condition with no universally accepted definition.

According to the official statement of the American Thoracic Society, mild asthma is characterized by minimal symptoms and risk, with little impairment and preserved lung function, not only in treatment-naive patients, but also in those who experience intermittent symptoms while receiving specific low-intensity asthma therapies, particularly short-acting β2-agonists (SABA) alone, without ICS [7].

This definition is indeed limited because it relies on retrospective symptom reporting and lung function tests that may be unreliable or not feasible in young children. The concept is further complicated by different phenotypes and endotypes, variable treatment responses, and frequent under-recognition or misperception of symptoms by children and caregivers [41].

Proposed criteria, such as daytime symptoms < 2/week, rare nocturnal symptoms, no more than one annual asthma attacks, and normal post-bronchodilator FEV₁, are difficult to apply consistently, especially in younger children. Reflecting these limitations, GINA 2025 advises caution and introduces the term “apparently mild asthma” to acknowledge that at any age low symptom frequency may mask significant underlying risk [11]. Prevalence estimates remain uncertain because no single definition reliably identifies all at-risk children, and even those appearing well-controlled may experience severe exacerbations, particularly when treated mainly with reliever-only SABA without adequate anti-inflammatory medication.

Several factors predict increased exacerbation risk, including frequent SABA use (> 2 inhalers/year), obesity, reduced lung function, elevated FeNO, blood eosinophilia, polysensitization, and previous exacerbations [42]. Tools such as the Asthma Control Test help standardize assessment of recent symptoms and reliever use, guiding treatment adjustments, although its four-week reference period may miss earlier exacerbations and underestimate future risk [43].

A major ongoing concern is the widespread use of SABA as monotherapy in children. While SABA provides quick and effective bronchodilation, its overuse is linked to higher risk of severe exacerbation and mortality [44]. In contrast, ICS reduce airway inflammation, enhance bronchodilator responsiveness, and decrease the likelihood of severe asthma attacks [45]. For this reason, GINA recommends that children take ICS whenever they use a SABA.

## GINA 2025 strategy report for the management of non-severe pediatric asthma

Effective ICS treatment is crucial for affordable asthma care and for reducing adverse effects, including those from SABA-only or systemic corticosteroids overuse [1]. In children not persistently symptomatic both under- and over-treatment carry relevant risks [46]. However, exposure to a trigger such as a viral respiratory tract infection, an allergen to which the child is sensitized, or atmospheric pollution can lead to airway inflammation in this population and cannot be ignored [47].

Based on GINA 2025 Report, key priorities include using ICS in an age-appropriate and targeted way, ensuring that anti-inflammatory treatment matches the child’s symptoms and developmental stage.

### Targeted and age-specific use of inhaled corticosteroids

#### Preschool children (< 5 years)

According to GINA 2025, children under 5–6 years with infrequent wheezing (**Step 1**) should preferably receive as-needed SABA monotherapy. However, due to concerns about SABA overuse, GINA recommends that in children with intermittent viral-induced wheezing and no interval symptoms who may not respond adequately to SABA, clinicians may consider short, intermittent courses of high-dose ICS at the onset of viral respiratory infections. This aligns with the observation that, at Step 1, airway responsiveness in 5-year-olds is indistinguishable from that of 6-year-olds, for whom GINA already recommends taking ICS whenever SABA is used [48]. Two additional considerations are fundamental in clinical practice: first, intermittent high-dose ICS should be used with caution and only when proper technique and adherence can be ensured; second, since post-viral lower airway symptoms usually resolve within 10 days [49], intermittent high dose ICS should generally not extend beyond this period, and if symptoms persist, escalation to Step 2 must be considered.

A 1-week course of nebulized beclometasone dipropionate (BDP, 400 µg twice daily) plus as-needed SABA has been shown to increase symptom-free days and improve cough in children aged 1–4 years presenting with mild or moderate wheezing episodes [50].

The leukotriene receptor antagonist (LTRA) montelukast is a potential therapeutic option, but GINA 2025 does not recommend its use for intermittent asthma in preschool children due to limited evidence.

In **Step 2**, daily low-dose ICS is recommended for children whose asthma is not well controlled or who have had ≥ 1 severe exacerbation in the previous year. However, it should be noted that the different ICS currently available have different minimum approved ages for use (see Table 2).

**Table 2:** ICS as single-component administered through nebulizer, DPI and pMDI available in Italy and corresponding age limits in the pediatric field as reported in the Italian SmPC (Summary of product Characteristics)

Active substance and pharmaceutical form	Age limit
Beclometasone dipropionate for nebulization	No age limit
Beclometasone dipropionate pMDI (100 mcg) “fine”	No age limit
Beclometasone dipropionate pMDI (100 mcg) “extra-fine”	≥ 18 years
Budesonide for nebulization	≥ 6 months
Budesonide pMDI (200 mcg)	≥ 6 years
Budesonide DPI (200 mcg)	≥ 6 years
Ciclesonide pMDI (80 mcg)	≥ 12 years
Flunisolide for nebulization	≥ 4 years
Fluticasone propionate for nebulization	≥ 4 years
Fluticasone propionate DPI	> 4 years
Fluticasone propionate pMDI	≥ 1 year
Mometasone fuorate DPI	≥ 12 years

Abbreviations: DPI: Dry Powder Inhaler; pMDI: pressurized Metered Dose Inhaler

For children with frequent viral-induced wheezing and interval asthma symptoms, as needed or episodic high-dose ICS may be considered, as strong evidence shows that intermittent ICS effectively prevents exacerbations [51].

A double-blind, double-dummy, randomized, parallel-group trial found that regular nebulized BDP was the most effective in increasing symptom-free days in preschool children with frequent wheezing [52]. BDP nebuliser suspension is also currently the only medication in Europe approved for the treatment of recurrent wheezing in children up to 5 years of age [53, 54].

#### Children 6–11 years

At **Step 1**, GINA 2025 recommends that children take a low-dose ICS whenever they use a SABA, ensuring that even those with mild or intermittent symptoms receive anti-inflammatory treatment alongside bronchodilation. This approach offers better protection against exacerbations since airway inflammation is present even in mild asthma [55].

The ICS/formoterol combination either as Anti-Inflammatory Reliever (AIR) only or as Maintenance-And-Reliever Therapy (MART), is not approved for use in this age group in any country, and GINA does not recommend it at Step 1.

For **Step 2**, daily low-dose ICS combined with as-needed SABA is recommended for symptom relief, effectively lowering the risk of severe exacerbations and enhancing overall asthma control compared with SABA alone [56].

In **Step 3**, after ensuring correct inhaler technique, adherence, and addressing modifiable risk factors, treatment escalation options include increasing ICS to a medium dose with as-needed SABA, switching to a low-dose ICS-LABA with as-needed SABA, or using MART with very low-dose ICS-formoterol.

Medium-dose ICS with as-needed SABA is the most robust and evidence-based approach, providing consistent symptom control, clear reductions in exacerbations, and alignment with approved pediatric indications [57].

Low-dose ICS-LABA with as-needed SABA has Evidence A according to GINA, based on the COMBO study in 158 children aged 6–16 years, which showed that combination therapy was non-inferior to the same dose of ICS alone in terms of the percentage of symptom-free days [58]. Moreover, the VESTRI trial, including 6,208 children aged 4–11 years, found no significant difference in exacerbation rates between ICS-LABA and ICS alone, suggesting that adding a LABA may not provide additional benefit in preventing severe exacerbations [59]. GINA prioritizes symptom-free days in pediatric studies, explaining the Evidence A rating, but this may overlook exacerbations, the most critical measure of asthma control and risk in children.

MART with very low-dose ICS-formoterol is rated Evidence B, based on post-hoc analyses of patients aged 4–80 years, which showed reduced exacerbations compared with the same dose of ICS-formoterol plus as-needed SABA or even higher-dose ICS in the overall population [60].

However, the study was not specifically powered for children, and evidence in those under 12 years is limited, making MART unsuitable as a standard option in this age group. Product labeling indicates that ICS-formoterol is approved for maintenance therapy in patients aged 6 years and older, but it is not intended for MART use in children under 12.

#### Adolescents > 12 years

For adolescents, GINA Track 1 recommends as-needed low-dose ICS–formoterol for symptom relief, including before exercise or allergen exposure. This approach applies to both **Step 1** and **Step 2** because it provides reliever and anti-inflammatory treatment in a single inhaler, decreases severe exacerbations and the need for oral corticosteroids, and allows easy step-up or step-down adjustments. In Italy, budesonide–formoterol may be used as MART only in adolescents aged ≥ 12 years and only at Step 3 or 4, given its rapid onset of action and suitability as both controller and reliever, but not as AIR-only [60].

Track 2 is an alternative when Track 1 is not suitable or when asthma is well controlled on the current therapy. At Step 1, this involves taking low-dose ICS whenever SABA is used, either via a combination inhaler or two separate inhalers. Although off-label in adolescents, this strategy is also supported by four RCTs showing that as-needed ICS with SABA offers protection against exacerbations comparable to daily ICS, while SABA-only therapy consistently leads to higher failure rates [61–64]. This approach helps avoid reliance on SABA alone and is particularly valuable for adolescents with poor adherence to daily ICS or in settings where ICS–formoterol is not available.

## Optimizing pediatric asthma management: challenges in inhaler technique

As highlighted in the GINA Report, incorrect inhalation technique, poor adherence and caregiver-related factors are major contributors to suboptimal asthma control in children [11]. Addressing these barriers requires structured, patient-centered strategies that integrate individualized device selection, regular assessment and reinforcement of inhalation technique, caregiver education, and supportive adherence tools, all coordinated by well-trained healthcare professionals to guide personalized treatment plans and optimize long-term clinical outcomes, as summarized in Table 3.

**Table 3:** Key barriers and proposed actions to optimize pediatric asthma management and inhaler use

Key area	Barriers	Proposed Actions
Incorrect inhalation technique	- High prevalence of improper technique (70–80% of children) - Poor coordination, incorrect inspiratory flow, not shaking pMDI - Confusion due to multiple device types	- Structured, patient-centered training for children and caregivers - Device selection tailored to age, dexterity, and preferences - Hands-on demonstrations, instructional videos, pictograms, QR-coded guides - Regular technique checks every 4–6 weeks - Consider nebulizers when inhalation skills are insufficient
Suboptimal adherence	- Low adherence rates (mean ICS adherence ~ 36%) - Forgetfulness, complex regimens, caregiver misunderstanding - “Pseudo-adherence” due to inability to detect empty inhalers	- Use of pMDIs with dose counters - Clear caregiver education on correct usage and monitoring - Simplify regimens when possible - Reinforce adherence at each visit
Healthcare professional training & caregiver engagement	- Limited HCP knowledge and practical skills on inhaler use - Caregiver concerns about ICS safety (“steroid-phobia”) - Mismatch between prescribed device and patient’s abilities or preferences	- Structured training programs for HCPs including hands-on practice - Empathetic, evidence-based communication to caregivers - Shared decision-making to select devices aligned with patient/caregiver preferences - Use of interactive tools (apps, videos, infographics) to enhance understanding and engagement

Abbreviations: ICS: inhaled corticosteroids; HCP: healthcare professional; pMDI: pressurized Metered Dose Inhaler

### Incorrect inhalation technique

**Fig. 1:**
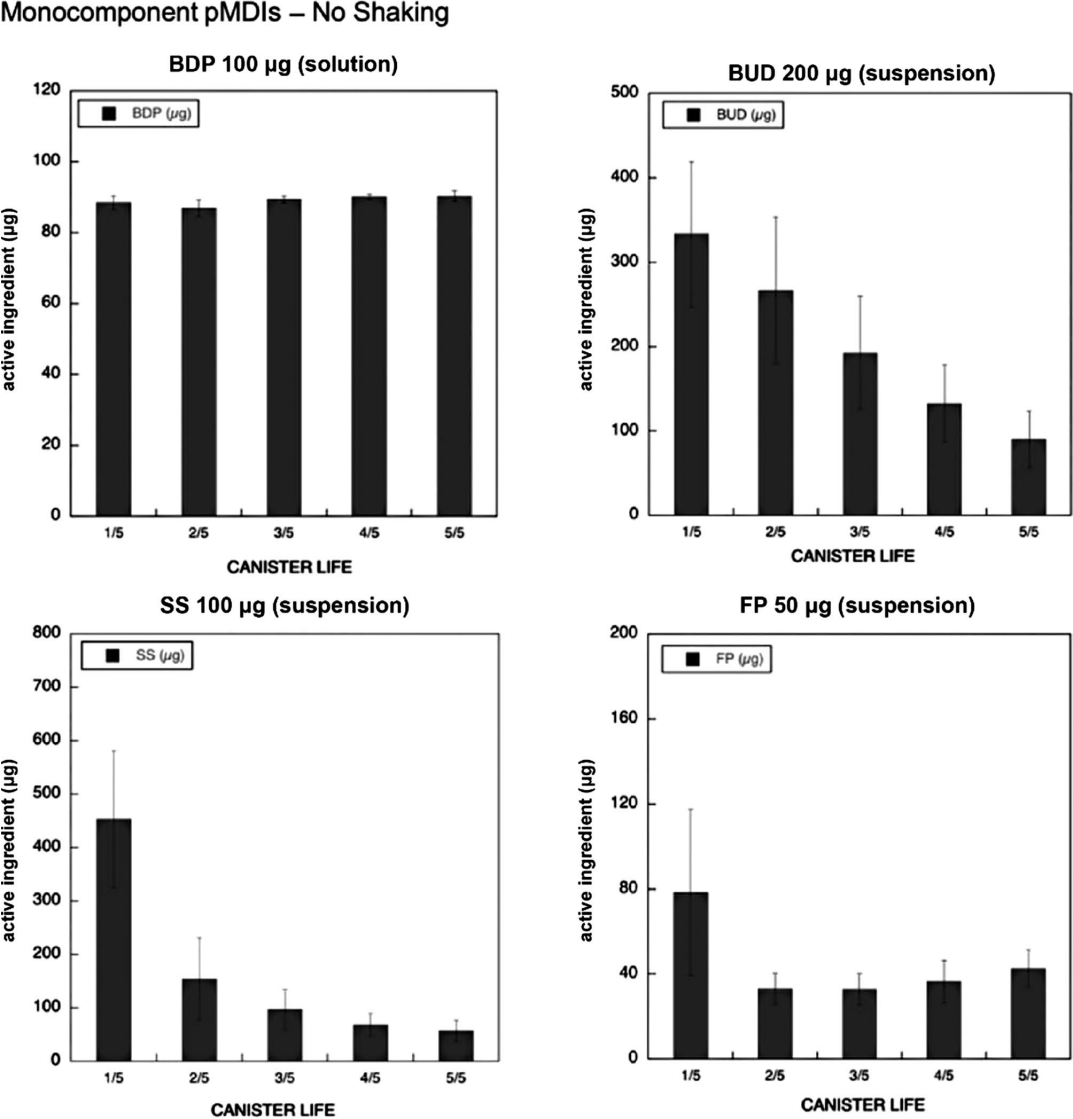
Emitted dose (µg) without shaking the canister at different level of canister content for monocomponent pMDIs. Reproduced modified with permission from Chierici et al. [68]. BDP: beclometasone dipropionate from Chiesi Farmaceutici S.p.A.; BUD: budesonide from Italchimici S.p.A.; FP: fluticasone propionate from GlaxoSmithKline S.p.A.; SS = salbutamol sulfate from GlaxoSmithKline S.p.A

Incorrect inhalation technique is a major barrier to optimal asthma control, with 70–80% of patients misusing inhalers, leading to poorer symptom management, higher exacerbation rates, and reduced efficacy [65, 66]. Common errors, especially in children, include insufficient inspiratory effort with DPIs, premature actuation or poor coordination with pressurized metered-dose inhalers (pMDIs), failure to remove the cap, and not shaking the canister, resulting in inconsistent and inadequate drug delivery [67]. Notably, failing to shake a suspension pMDI can cause significant variability in the delivered dose, whereas solution formulations generally remain stable (Fig. 1) [68]. Improper inhaler technique has significant clinical consequences; errors such as not opening the inhaler correctly or failing to shake the canister are associated with a 47% increased risk of uncontrolled asthma [67], and over half of pediatric patients do not shake their pMDI before use [69–71]. For children who cannot use inhalers reliably, nebulizers provide a practical and effective option, as the caregiver prepares the dose and the child simply breathes normally—essentially, they “*just breathe!*”

### Suboptimal adherence

Adherence to inhaled controller therapy remains a major obstacle in children with wheezing and asthma, with average ICS adherence around 36% and only 15% achieving the commonly used threshold of ≥ 75% adherence [72]. Poor adherence is influenced by complex dosing regimens, limited caregiver understanding, forgetfulness, and difficulties coordinating administration with daily routines, and is linked to higher exacerbation risk and hospitalizations [73–75].

A common but often overlooked factor in poor adherence is the inability to recognize when a pMDI is empty. Patients and caregivers may mistakenly believe the inhaler still contains medication because a visible spray persists, even after the active ingredient is exhausted, leading to either premature disposal or prolonged use of empty canisters—a phenomenon known as “pseudo-adherence”, which reflects inadvertent incorrect use of prescribed medication [76].

Tracking treatment adherence and ensuring consistent drug delivery is particularly critical in children who rely on caregivers and may not recognize early signs of treatment failure. The integration of dose counters into pMDIs provides a clear and quantitative indication of remaining doses, enabling accurate monitoring, timely replacement, and reduced risk of overuse, underuse, and pseudo-adherence, while also improving confidence, satisfaction, and aligning with patient preferences for practical, easy-to-use inhaler features [77–79].

### Healthcare professional training, caregiver education, and shared decision-making for choice of inhaler device

Effective asthma management depends on well-trained healthcare professionals (HCPs) who can accurately diagnose, prescribe therapy, and educate patients and caregivers. Many HCPs, however, lack sufficient knowledge and practical skills, particularly in correct inhaler technique, leading to suboptimal drug delivery, increased exacerbations, and poor asthma control [80, 81].

Targeted, comprehensive educational programs combining verbal instruction, hands-on demonstrations, and patient-centered training improve HCPs’ knowledge, skills, and confidence, enabling them to address adherence barriers, demonstrate correct inhaler use, and provide effective asthma education [82–84]. Such interventions are particularly valuable for addressing caregiver “steroid-phobia”, as concerns about impaired growth, systemic side effects, and long-term consequences often lead to premature discontinuation or underdosing of ICS, despite evidence supporting the safety of low-to-moderate doses, ultimately resulting in significantly poorer asthma control in children [85, 86]. However, it is important to recognize that not all ICSs are pharmacologically equivalent, as their activation pathways and systemic metabolism differ. For example, BDP has a metabolic profile that makes it less likely to cause adrenal insufficiency [87–90] and reduces the potential for drug–drug interactions [90–92].

Overcoming steroid-phobia and promoting adherence in pediatric asthma requires empathetic, evidence-based communication, supported by visual and interactive tools, along with structured HCP training. This approach facilitates shared decision-making in inhaler selection, actively engaging caregivers and patients in choosing devices suited to their abilities, preferences, and lifestyle [93–97]. The GINA report emphasizes that selecting the most appropriate inhaler for each patient is crucial at every age, with careful consideration of patient preference and satisfaction. Although device choice is often influenced by local prescribing habits and cultural familiarity, incorporating patient-centered decision-making enhances adherence and clinical outcomes and is recommended as a core element of asthma management.

In Italy, nebulizers remain widely used, accounting for approximately 45% of inhaled medication sales, particularly in children, reflecting both physician prescribing habits and caregiver familiarity [98, 99]. Their popularity is supported by clear physiological and practical advantages, as nebulizers require only normal breathing and no patient coordination, making them especially suitable for infants, young children, uncooperative patients, or those with impaired consciousness [100, 101]. Surveys and studies across different regions confirm this trend [102–105]. For example, among children aged 1–5 years, 81% in the USA, 55% in Northern Europe, and 67% in Southern Europe used nebulizers, compared with 25%, 51%, and 34% who used pMDIs with spacers, respectively [102]. This preference should be respected in clinical practice, as robust evidence demonstrates equivalent clinical outcomes with pMDIs, DPIs, or nebulizers when the device is used correctly [106]. Finally, the use of nebulisers represents a viable option to reduce the carbon footprint associated with inhaled therapies [107].

Therefore, aligning device choice with patient and caregiver familiarity, while ensuring correct technique through training and education, is essential to optimize asthma control, adherence, and long-term clinical outcomes in pediatric populations.

## Conclusions

Pediatric wheezing and mild asthma represent a heterogeneous spectrum of disease, characterized by variable trajectories, risk profiles, and treatment responses, requiring a structured, patient-centered approach. Early identification of children at risk for persistent asthma—through careful clinical evaluation, phenotypic characterization, biomarker assessment (blood eosinophils, FeNO), and validated predictive tools such as API/mAPI—enables timely, individualized interventions aimed at modifying disease progression and preserving lung function.

The primary aim of this Expert Opinion paper is to translate the GINA 2025 strategy report into real-world pediatric practice in Italy, addressing challenges such as early patient identification, risk stratification, personalized treatment strategies, and appropriate device selection to improve long-term disease control and overall patient outcomes.

The implementation of GINA 2025 recommendations in a specific context should consider several practicalissues, including age-appropriate device selection, ongoing caregiver education, and reinforcement of correct inhaler technique. Actively addressing adherence barriers and promoting shared decision-making can enhance asthma control, reduce exacerbations, and improve long-term respiratory outcomes [108]. Ultimately, bridging the gap between evidence-based recommendations and clinical practice requires not only medical expertise but also effective communication, active caregiver engagement, and system-level support to ensure truly personalized care for each child with wheezing or mild asthma.

## Supplementary Information

Below is the link to the electronic supplementary material.

Supplementary Material 1

## Data Availability

Not applicable.

## Abbreviations

AIR: Anti-Inflammatory Reliever
API: Asthma Predictive Index
BDP: Beclometasone Dipropionate
BUD: Budesonide
DPI: Dry Powder Inhaler
EBC: Eosinophil Blood Count
ERS: European Respiratory Society
FEF: Forced Expiratory Flow
FeNO: Fractional exhaled Nitric Oxide
FEV: Forced Expiratory Volume
FVC: Forced Vital Capacity
FP: Fluticasone Propionate
GINA: Global Initiative for Asthma
HCP: Healthcare Professional
ICS: Inhaled Corticosteroids
LABA: Long-Acting Beta-Agonist
LTRA: Leukotriene Receptor Antagonist
mAPI: Modified Asthma Predictive Index
MART: Maintenance And Reliever Therapy
PARS: Pediatric Asthma Risk Score
pMDI: Pressurized Metered Dose Inhaler
RCT: Randomized Controlled Trial
SABA: Short-Acting Beta-Agonist
SmPC: Summary of Product Characteristics
sIgE: specific Immunoglobulin E
SPT: Skin Prick Test
SS: Salbutamol Sulfate
