# Supplementary material for: An expert opinion on the management of pediatric patients with wheezing and mild asthma: translating 2025 GINA strategy report into clinical practice in Italy
Source: Ital J Pediatr. 2026 Feb 4;52:34. doi: 10.1186/s13052-026-02206-9 (PMC12958778; doi:10.1186/s13052-026-02206-9)
Supplement: Supplementary file 1 — Supplementary Material 1 [file 13052_2026_2206_MOESM1_ESM.pdf]

PARTIES:

1. **INFORMA UK LIMITED** (Licensor); and
2. **Francesca Santamaria** (Licensee).

Thank you for your recent permission request. Some permission requests for use of material published by the Licensor, such as this one, are now being facilitated by PLSclear.

Set out in this licence cover sheet (the **Licence Cover Sheet**) are the principal commercial terms under which Licensor has agreed to license certain Licensed Material (as defined below) to Licensee. The terms in this Licence Cover Sheet are subject to the attached General Terms and Conditions, which together with this Licence Cover Sheet constitute the licence agreement (the **Licence**) between Licensor and Licensee as regards the Licensed Material. The terms set out in this Licence Cover Sheet take precedence over any conflicting provision in the General Terms and Conditions.

Licence Terms

Licence Date:	27/11/2025
PLSclear Ref No:	111963

The Licensor

Company name:	INFORMA UK LIMITED
Address:	4 Park Square<br>Milton Park<br>Abingdon<br>Oxon<br>OX14 4RN<br>GB

The Licensee

Licensee Contact Name:	Francesca Santamaria
Licensee Address:	ITALIA<br>ETHOS SRL VIA BERNA 9<br>ROMA<br>00144<br>Italy

Licensed Material

title:	Consequences of not-shaking and shake-fire delays on the emitted dose of some commercial solution and suspension pressurized metered dose inhalers

ISBN/ISSN:	10.1080/17425247.2020.1767066
publisher:	INFORMA UK LIMITED
Issue Date	2020
Volume number	17
Issue number	7
Are you requesting permission to reuse the cover of the publication?	No
Article Title	Consequences of not-shaking and shake-fire delays on the emitted dose of some commercial solution and suspension pressurized metered dose inhalers
Article URL	https://doi.org/10.1080/17425247.2020.1767066
Figure number & title	Emitted dose without shaking the canister (monocomponent pMDIs)
Page numbers	1025-1039
Name of illustrator	Not specified (authors' own figure)
Are you the author of the content that you are requesting to reuse?	No
Is the content you are requesting available through an Open Access Licence?	Yes
Additional Information	This request concerns the reuse of Figure 1 from the original publication.<br>The figure has been slightly adapted for layout and formatting purposes only;<br>the scientific content and data are unchanged.<br><br>Although one of the authors of the 2020 ar
Will you be changing or editing the image?	Yes
Will it be cropped?	No
Full details of how it will be altered	The figure has been adapted with minor graphic changes (layout, spacing, removal of caption)<br>without altering any data or scientific content.<br>No cropping has been performed.

For Use In Licensee's Publication(s)

usage type	Journal or Magazine Article-Electronic Journal
Article author	Francesca Santamaria1*, Eugenio Baraldi2,3, Luca Cavalieri4, Renato Cutrera5, Stefania La Grutta6, Giorgio Piacentini7, Gherardo Siscaro8
Article title	An Expert Opinion on the Management of Pediatric Patients with Wheezing and Mild Asthma: Translating 2025 GINA Strategy Report into Clinical Practice in Italy
Issue date	2025-12-31
Language	English
Open Access	Green
Other territory	ITALY
Publication title	An Expert Opinion on the Management of Pediatric Patients with Wheezing and Mild Asthma: Translating 2025 GINA Strategy Report into Clinical Practice in Italy

Publisher	ITALIAN JOURNAL OF PEDIATRIC
Retail or subscription price	1000
Unlimited circulation?	Yes

Rights Granted

Exclusivity:	Non-Exclusive
Format:	Online Edition
Language:	English
Territory:	ITALY
Duration:	Lifetime of Licensee's edition
Maximum Circulation:	Maximum web circulation: Unlimited copies

Additional Terms:

© 2020 The Author(s). Published by Informa UK Limited, trading as Taylor & Francis Group.
This is an Open Access article distributed under the terms of the Creative Commons Attribution-NonCommercial NoDerivatives License (http://creativecommons.org/licenses/by-nc-nd/4.0/), which permits non-commercial re-use, distribution, and reproduction in any medium, provided the original work is properly cited, and is not altered, transformed, or built upon in any way.

Fees Apply as the reuse is being transformed

All reasonable efforts must be made to contact the author(s) to notify them of your intended use.

The permission covers a onetime use and any further use requires additional permission.

This license does not cover any use of the Licenced Content other than as expressly authorized under this agreement is prohibited. Without limiting the foregoing, the Licensed Content may not be used in a custom publishing program, database, individual chapters’ sales, or input into any artificial intelligence or large language model tool, system, or model (“AI Tools”).

Any use of the Licenced Content with AI Tools requires a separate written license from Taylor & Francis.

Payment Details

Fee Payable:	£133.25 [+ VAT if applicable]
Payment Terms:	Strictly 30 days from date of Licence

GENERAL TERMS AND CONDITIONS

1. Definitions and Interpretation

1.1 Capitalised words and expressions in these General Terms and Conditions have the meanings given to them in the Licence Cover Sheet.

1.2 In this Licence any references (express or implied) to statutes or provisions are references to those statutes or provisions as amended or re-enacted from time to time. The term **including** will be construed as illustrative, without limiting the sense or scope of the words preceding it. A reference to in **writing** or **written** includes faxes and email. The singular includes the plural and vice versa.

2. Grant of Rights

2.1 Subject to payment by Licensee of the Licence Fee in accordance with paragraph 3 below, Licensor grants to Licensee the non-exclusive right to use the Licensed Material as specified in the Licence Cover Sheet.

2.2 The rights licensed to Licensee under this Licence do not include the right to use any third party copyright material incorporated in the Licensed Material. Licensee should check the Licensed Material carefully and seek permission for the use of any such third party copyright material from the relevant copyright owner(s).

2.3 Unless otherwise stated in the Licence Cover Sheet, the Licensed Material may be:

2.3.1 subjected to minor editing, including for the purposes of creating alternative formats to provide access for a beneficiary person (provided that any such editing does not amount to derogatory treatment); and/or

2.3.2 used for incidental promotional use (such as online retail providers’ search facilities).

2.4 Save as expressly permitted in this Licence or as otherwise permitted by law, no use or modification of the Licensed Material may be made by Licensee without Licensor's prior written permission.

3. Payment

3.1 Licensee must pay to Licensor the Licence Fee by means of either credit card or on receipt of an invoice, as selected by Licensee during the licence application process via the PLSclear service.

3.2 If payment is by invoice, Licensee agrees to pay the Licence Fee in full by no later than the payment date specified in the relevant invoice.

4. Copyright Notice and Acknowledgement

4.1 Licensee must ensure that the following notices and acknowledgements are reproduced prominently alongside each reproduction by Licensee of the Licensed Material:

4.1.1 the title and author of the Licensed Material;

4.1.2 the copyright notice included in the Licensed Material; and

4.1.3 the statement "Reproduced with permission of the Licensor through PLSclear."

5. Reversion of Rights

5.1 The rights licensed to Licensee under this Licence will terminate immediately and automatically upon the earliest of the following events to occur:

5.1.1 the Licence Fee not being received by Licensor in full by the payment date specified in the relevant invoice;

5.1.2 the Licensed Material not being used by Licensee within 18 months of the Licence Date;

5.1.3 expiry of the Licence Duration; or

5.1.4 the Maximum Circulation being reached.

6. Miscellaneous

6.1 By using the Licensed Material, Licensee will be deemed to have accepted all the terms and conditions contained in this Licence.

6.2 This Licence contains the entire understanding and agreement of the parties relating to its subject matter and supersedes in all respects any previous or other existing arrangements, agreements or understandings between the parties whether oral or written in relation to its subject matter.

6.3 Licensee may not assign this Licence or any of its rights or obligations hereunder to any third party without Licensor's prior written consent.

6.4 This Licence is governed by and shall be construed in accordance with the laws of England and Wales and the parties hereby irrevocably submit to the non-exclusive jurisdiction of the Courts of England and Wales as regards any claim, dispute or matter arising under or in relation to this Licence.
